# Inter-professional collaboration of nurses and midwives with physicians and associated factors in Jimma University specialized teaching hospital, Jimma, south West Ethiopia, 2019: cross sectional study

**DOI:** 10.1186/s12912-020-00426-w

**Published:** 2020-04-26

**Authors:** Eneyew Melkamu, Solomon Woldemariam, Abera Haftu

**Affiliations:** 1 School of nursing and midwifery, college of health sciences, Jimma University, P.O.Box:378, Jimma, Ethiopia; 2 Department of midwifery, college of health sciences, Mekelle University, P.O.Box:231, Mekelle, Ethiopia

**Keywords:** Inter-professional collaboration, Nurse-physician, Midwife-physician

## Abstract

**Background:**

Inter-professional collaboration between professionals is crucial in health care where most of the activities are undertaken in a team. One of these collaborations is the collaboration of nurses and midwives with physicians. The main objective of this study was to assess interprofessional collaboration of nurses and midwives with physicians and associated factors at Jimma University specialized teaching hospital from March 20 to April 8, 2019.

**Methods:**

An institution-based cross-sectional study was conducted among 358 nurses and 52 midwives who are working in Jimma University Specialized teaching hospital using a structured self-administered questionnaire. Study units were selected by simple random sampling using the lottery method. The result was summarized using descriptive statistics and statements. The level of significance was set at a *p* < 0.05.

**Result:**

The overall response rate was 99.76%. Around two-third, 66.7% (*n* = 273) of participants had a satisfactory inter-professional collaboration with physicians and 238 (58.2%) had good relationship with physicians. Again 234 (57.2%) of participants had a favorable attitude towards interprofessional collaboration with physicians. Moreover, statistical significance was obtained on the relationship of participants with physicians (*p* = 0.000), the experience of disruptive behavior (*p* = 0.000), attitude towards interprofessional collaboration with physicians (p = 0.000) and occupational status (*p* = 0.001).

**Conclusion:**

The majority of the participants had a satisfactory inter-professional collaboration with physicians and four of the many possible factors under consideration were finally found statistically significant. Again, it was revealed that nurses and midwives did not significantly differ in their inter-professional collaboration with physicians.

## Background

Inter-professional collaboration is a process involving jointly advantageous active participation between independent professionals in which each member of the health care team has knowledge and skills that underwrite to the care provided and their relationships are governed by negotiated shared norms and visions [1].

Health care provision needs many interactions and collaborations between different healthcare professionals with varying levels of education and professional qualifications [2]. Effective collaboration of nurses and midwives with physicians who work together in a dynamic and complex care environment helps to enhance patient well-being, quality of care, and provider satisfaction [3–5].

As care needs become more sophisticated, it is less probable that one health care provider alone can meet them and the more important it is to collaborate [6]. While collaborative working between health care professionals is largely promoted as the goal of present-day hospitals, it is difficult to achieve in the real setting as professional rivalries and deep-seated philosophical differences of care provision generate significant pressures [7].

The World Health Organization (WHO) Framework for interprofessional education and collaboration recognized that many health systems and health professionals throughout the world are disjointed and harassed to manage unmet health needs [8]. Though nursing and medicine are among the disciplines that work observantly and share a common pledge to patient well-being, a common type of struggle in health facilities is that between nurses and physicians which is caused by lack of daily interaction and teamwork [9].

It is demonstrated in two different studies that more than half of nurses and midwives exhibited non-collaborative behaviors and generally had not a good collaboration with physicians [10, 11]. Another study conducted in Iran indicated that near half (48%) of nurses reported their remarks concerning the patient’s health are ignored by physicians [12]. In Ethiopia, a study conducted at Felegehiwot and Gondar referral hospitals showed that more than one-third of nurses (41%) rate their collaboration as poor and only (3%) of nurses rate it as excellent [13].

Different pieces of literature had concluded that inter-professional collaboration of nurses and midwives with physicians is affected by their age, year of experience, sex, educational status, working area and attitude towards collaboration with physicians [9, 10, 14–16]. Again hospital rules and regulations, different care plans of the professionals, lack of adequate midwives and nurses at the administrator level and lack of adequate time are also found to be associated factors [17, 18]. Those research articles dealing with factors associated with inter-professional collaboration (IPC) of nurses and midwives with physicians have reached different findings on which factors depend on the effective collaboration or can be achieved through improved collaboration between those professionals.

In Ethiopia, the proportion of nurse, midwife, and doctor to patient and qualification levels are showing tremendous improvements as seen from the first phase of the health sector development plan (HSDP) till the end of the first health sector transformation plan [19]. However, at the end of HSDP II, prior to the major human resource reforms, Ethiopia ranked in the lowest quintile among African nations in terms of density of healthcare personnel, with 0.3 physicians and 2 nurses per 10,000 population and only 2 midwives per health center. Most of the nurses and midwives are also at diploma and bachelorate degree level working in all health facilities including, different level of hospitals, health centers, health posts and at leadership positions at various levels. There was also a problem of uneven distribution of the limited health workforce among and within districts and an inappropriate use of available skills with the less collaborative settings [19–22].

Like most developing countries, Ethiopia has inadequate healthcare resources but with a vast healthcare needs and with hospitals which are congested with patients placing tremendous pressure and heavy workload to the health care providers [13]. Apart from the government’s effort to increase the number and quality of healthcare institutions, it is imperative to have a positive collaboration of nurses and midwives with physician though is not assessed adequately.

To the best of the author’s knowledge, regarding nurse-physician inter-professional collaboration particularly between midwives and physicians are not well investigated and insufficient to inform policymakers and other concerned bodies in Ethiopia. The few works of literature available in Ethiopia also did not assess important factors especially at the interpersonal level like attitude and behavioral aspects. Therefore, this study tried to fill the gap and assessed the problem better.

## Methods

### Aim of the study

1. To assess inter-professional collaboration of nurses and midwives with physicians at Jimma university specialized teaching hospital, Jimma, Southwest Ethiopia
2. To determine factors associated with the interprofessional collaboration of nurses and midwives with physicians at Jimma university specialized teaching hospital, Jimma southwest Ethiopia

### Study area, period and population

This institution based cross-sectional study was conducted from March 29 to April 12, 2019 G. C in Jimma University Specialized teaching Hospital which is located in Jimma town 352 km southwest of the capital of Ethiopia, Addis Ababa. Currently, it is the largest and the only teaching and referral hospital in the southwestern part of the country, providing services for approximately 15,000 inpatient, 160,000 outpatient attendants, 11,000 emergency cases and 4500 deliveries in a year coming to the hospital from the catchment population of about 15 million people.

It has 1600 staff of whom 648 are nurses and midwives (566 nurses and 82 midwives). The hospital also has 800 beds and provides many health care services in the gynecology and obstetrics, internal medicine, pediatrics, emergency, radiology, surgery, and other departments.

The study population was nurses and midwives working in Jimma University Specialized teaching hospital who met the inclusion criteria (one, nurses and midwives with length of service half a year and above were included as they are considered to have more experience of working with physicians in addition to being considered full employees by the civil service law of Ethiopia and two, available during the study period was also an inclusion criteria).

### Sample size and sampling procedure

The sample size (n) was calculated using the formula to estimate a single population proportion:

n = [(Zα/2)2 p (1-p)/d2]. Then, the minimum sample size: n = (1.96)2 (0.41) (0.59) / (0.05)2 = 371.71 ≈ 372 taking *p* = 0.41 from a previous study done in North west Ethiopia [13].

Adding 10% for non-response rate, the final sample size was calculated to be: *n* = 372 + 37.2 ≈ 410. Using population proportion formula: ni = Ni × n/N, number of nurses = 566 × 410/648 = 358.12 ≈ 358 and number of midwives = 82 × 410/648 = 51.88 ≈ 52. Therefore, a total of 358 nurses and 52 midwives were included in the study.

A stratified sampling technique was used to select the study population. The study population was stratified by profession to nurses and midwives and the sample was taken from each stratum proportionally. Individual participants were selected using simple random sampling with a lottery method to attain the final sample size. A list of nurses and midwives from each ward was used as a sampling frame.

### Operational definitions

#### Favorable attitude towards interprofessional collaboration with a physician

Higher factor median score (55 and above) on overall score of the adapted Jefferson scale of attitude towards nurse-physician collaboration.

#### Unfavorable attitude towards interprofessional collaboration with a physician

Lower factor median score (below 55) on overall score of the adapted Jefferson scale of attitude towards nurse-physician collaboration.

#### Satisfactory interprofessional collaboration

Higher factor score (10 and above) on the inter-professional collaboration measuring items.

#### Unsatisfactory interprofessional collaboration

Lower factor score (below 10) on the inter-professional collaboration measuring items.

#### Relationship with physicians

Relationship with physicians was rated by individual participantsas having good relationship or not with physicians.

### Data collection procedure

#### Data collector

Data collection was facilitated by five trained data collectors who are Bachelor science degree (BSc) holders.

#### Instrument

Inter-professional collaboration of nurses and midwives with physicians was assessed using 14 items with options “YES” or “NO” that was prepared by reviewing related works of literature. The socio- demographic characteristics were assessed using close ended questions that were prepared after relevant literatures were reviewed. I.e. almost all references mentioned at the end of this manuscript were used to develop those inter-professional collaboration and socio- demographic items. The attitudes of nurses and midwives were assessed by the adapted version of Jefferson Scale of Attitudes toward Nurse Physician Collaboration. The final version of the JSANPC contains 15 items answered on a 4-point Likert-type scale from (1“strongly disagree” to 4“strongly agree”). A higher total score reflects a more positive attitude toward physician-nurse collaborative relationships.

The instrument has known factors which were identified as: * ‘shared education and teamwork’, ‘caring as opposed to curing’, ‘nurse’s autonomy’ and ‘physician’s authority’. The tool was originally developed by Hojat and Herman in 1985 and was modified in 2003 by Hojat et al. [23].

This tool was supported by psychometric evidence including construct validity and internal consistency reliability that can be used as a research tool in western countries. According to Hussein SZ1, Fatin Amira Ahmad and S. Hawa M. Noh (2018) the tool has good internal consistency with a Cronbach’s alpha coefficient reported of 0.87 [24]. In the current study, it was found to have a Cronbach’s alpha coefficient of 0.72 which is within the acceptable range. To check whether it works in Ethiopia, a pre-test was conducted by a study done in North West Ethiopia in Goba referral hospital and finally confirmed that the tool can be applied in the Ethiopian context [13]. This tool (JSANPC) was preferred from other psychometrically supported instruments available for measuring physician-nurse collaborative relationships as it was checked (applied) in Ethiopian context and confirmed to be valid and reliable to be used in Ethiopian studies.

#### Data collection method

Data was collected by administering a written questionnaire to study participants which was prepared originally in English and then translated into Affann Oromo and Amharic (the local languages in the study area) by a language expert in all those three languages. Then, it was translated back to English to keep its consistency and validity.

### Data quality control

In order to maintain the quality of the data, a pretest was done on 41 (10%) of nurses and midwives in a different hospital found in Jimma city, South West Ethiopia and necessary modifications including wordings were made on the questionnaire before it was applied on the study population. Furthermore, the questionnaires were checked for completeness before data entry. The data was interred in software called Epi data version 4.2 to point out errors made during data collection automatically then transferred to Statistical Packages for Social Sciences (SPSS) version 23. Furthermore, training was given to data collectors and supervisors and the overall data collection process was monitored by supervisors**.**

### Data processing and analysis

The collected data was entered into epi data version 4.2 and exported to SPSS version 23 for cleaning and further analysis. Binary and multivariable logistic regressions were run to assess the association between the dependent and independent variables. Again, a *p*-value of less than 0.25 and 0.05 in binary and multivariable logistic regression respectively were considered as significant at a 95% confidence level. Mann Whitney U test was used to evaluate the difference between nurses and midwives inter-professional collaboration with physicians. Results were presented by frequency tables, percentages, measures of central tendency and dispersion and statements.

## Result

### Socio-demographic characteristics of study participants

A total of 410 participants, 358 (87.3%) nurses and 52 (12.7%) midwives were involved in the study. There was one incomplete questionnaire yielding a response rate of 99.76%. Of the total respondents, 234 (57.21%) were males and 175 (42.79%) were females. The mean age of the respondents was 32.14 (SD ± 7.12) years. The other socio-demographic characteristics of study participants are mentioned in Table 1 below.

**Table 1:** Socio-demographic characteristics of respondents (*n* = 409) in Jimma University Specialized Teaching Hospital, Jimma, South West Ethiopia, 2019

Characteristics	Frequency	Percentage
Religion		
Orthodox	196	47.9
Muslim	125	30.6
Protestant	77	18.8
Catholic	9	2.2
Others	2	0.5
Ethnicity		
Oromo	185	45.2
Amhara	102	24.9
Tigrian	23	5.6
Gurage	25	6.1
Others	74	18.1
Marital status		
Single	149	36.4
Married	252	61.6
Divorced	7	1.7
Widowed	1	0.2
Age		
20–29	166	40.5
30–39	183	44.6
40–62	69	14.9
Educational status		
Diploma		
Nurse	81	22.7
Midwife	6	11.5
BSc degree		
Nurse	273	76.5
Midwife	46	88.5
MSc degree and above		
Nurse	3	0,8
Midwife	_	_

### Work-related and other characteristics of the participants

Concerning the occupational status of the respondents, most 393 (96.1%) were staff nurses and midwives (ordinary staffs) and 16 (3.9%) were head nurses and midwives. The mean year of services for the participants was 6.93 (SD ± 5.93) years. The result again showed that most of, 332 (81.17%) of the participants had 0.5 to 10.5 years of service. The respondents also give services to an average of 34.36 (SD ± 15.68) patients per day.

**Fig. 1:**
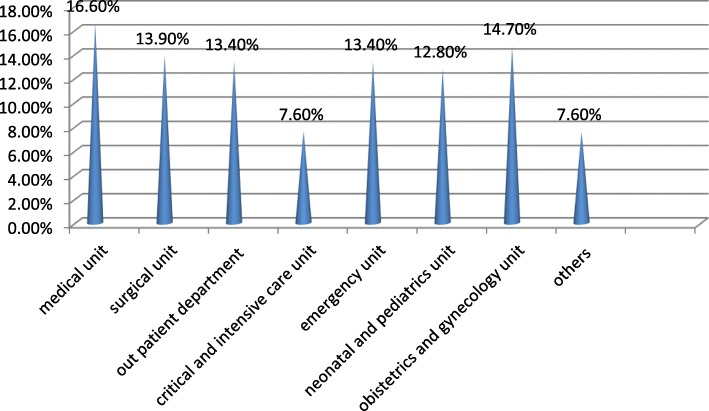
Working area of respondents (*n*=409) in Jimma university Specialized teaching hospital, Jimma, south west Ethiopia, 2019

As shown in figure one below, 68 (16.6%) of the participants work in the medical units and 60 (14.7%) of them work in the gynecology and obstetrics units (Fig. 1).

Regarding the relationship between nurses and midwives with physicians, 238 (58.2%) participants had good relationship with physicians. Again, around four out of 10, 170 (41.6%) of the respondents reported that they experience at least one disruptive behavior in their working area. In this study, 264(64.5%) of the respondents said that the hospital rules and regulations help them to do in collaboration with physicians.

### The attitude of respondents towards inter-professional collaboration with physicians

The statistical measure which was used to categorize the score of nurses and midwives into “favorable” and “unfavorable” attitude was the median value. It was because the distribution of scores on the scale was not symmetrical (not normally distributed). The median score on the overall scale of JSANPC was 55 (IQR ± 4.0).

More than five out of 10 234 (57.2%) of the participants had favorable attitudes towards collaboration with physicians and the rest 175 (42.8) of them had an unfavorable attitude towards collaboration with physicians.

### The inter-professional collaboration of nurses and midwives with physicians

Around two-third, 273 (66.7%) of study participants had a satisfactory inter-professional collaboration with physicians and the remaining 136 (33.3%) were found to have unsatisfactory inter-professional collaboration with physicians. Furthermore, the median score of participants on collaborative behavior measuring items was 11 (IQR ± 3.0). Considering them separately, 234 (65.5%) of nurses and 39 (75%) of midwives had a satisfactory inter-professional collaboration with physicians.

Table two below illustrated the response of participants to the inter-professional collaboration with physicians measuring items. As seen from the table, almost nine out of 10 respondents, 365 (89%) perform greeting with physicians. In contrast, less than half of participants respond “yes” to the item “trying to prevent medical care accidents together with physicians”, 194(47.4%) (Table 2).

**Table 2:** Responses of participants on the inter-professional collaboration with physicians measurement items (*n* = 409) in Jimma University specialized teaching hospital, Jimma, south west Ethiopia, 2019

Characteristics	Yes	No
Do you greet with physicians	364 (89%)	45 (11%)
Do you discuss patient’s problem with physicians and give solutions?	336 (82.2%)	73 (17.8%)
Do you hold discussions to resolve differences of opinion with physicians in the event of discrepancy about direction of patient care?	315 (77.0%)	94 (23.0%)
Do you plan together with physicians about patient needs when patient is to be admitted or discharged to the hospital?	318 (77.8%)	91 (22.2%)
Do you set together the future directions of patient care with physicians?	309 (75.6%)	100 (24.4%)
Do you try to prevent medical care accidents together with physicians?	194 (47.4%)	215 (52.6%)
Do you share each other information about a patient’s condition and reaction to treatment with physicians?	307 (75.1%)	102 (24.9%)
Do you support each other with physicians during patient care?	312 (76.3%)	97 (23.7%)
Do you exchange informations and opinion about matters related to work with physicians?	279 (68.2%)	130 (31.8%)
Do you show concern for one another with physicians when they are in need of your help?	300 (73.3%)	109 (26.7%)
Do you share each other’s opinions with physicians to resolve problems related to patient care?	304 (74.3%)	105 (25.7%)
Do you take into account each other’s schedule when you make plan to give care for the patient with physicians?	291 (71.1%)	118 (28.9%)
Do you always consult physicians when you need them?	309 (75.6%)	100 (24.4%)
Do you respond to each other’s call when you are in need during patient care?	361 (88.3%)	48 (11.7%)

### Difference between nurses and midwives in inter-professional collaboration with physicians

The non-parametric equivalent of independent samples T-test (Mann Whitney U test) was used to assess the difference as the distribution of the data deviates from normality. This test revealed that there was a difference between nurses and midwives in their inter-professional collaboration with physicians (Mann Whitney U = 6793.0, Z = − 3.157, *p* = 0.002). But, it is a bivariate result that the test showed and finally it was confirmed that profession was not a significant factor in the multivariable logistic regression which is stated below. Hence, there was no significant difference in IPC of nurses and midwives with physicians.

### Factors associated with the inter-professional collaboration of nurses and midwives with physicians

In the final model (multivariable logistic regression), relationship with physicians, attitude towards interprofessional collaboration with physicians, occupational status and experience of disruptive behaviors were found statistically significant. As shown in Table 3 below, the odds of those participants who had good relationship with physicians increases by more than three folds to have satisfactory IPC with physicians when compared with those who did not have good relationship with physicians (*p* = 0.000, AOR = 3.48, CI: 2.077–5.825).

**Table 3:** Regression output of factors associated with inter-professional collaboration of participants with physicians (n = 409) in Jimma university specialized teaching hospital, Jimma, south west Ethiopia, 2019

Variable	Frequency		COR (95% CI)	AOR (95% CI)	*p*-value
	Satisfactory inter-Professional collab- Oration	Unsatisfactory inter-Professional collab- Oration			
Profession					
Nurse	234 (65.5%)	123 (34.5%)	0.634 (0.326–1.233)	0.741 (0.338–1.624)	0.454
Midwife	39 (75%)	13 (25%)		**1**	
Hospital rules and regulation are helpful to collaborate					
No	82 (56.6%)	63 (43.4%)	0.497 (0.325–0.761	0.633 (0.384–1.045)	0.074
Yes	191 (72.3%)	73 (27.7%)		**1**	
Good relationship with physicians					
Yes	187 (78.6%)	51 (21.4%)	3.624 (2.355–5.576)	3.478 (2.077–5.825)	0.000*
No	86 (50.3%)	85 (49.7%)		**1**	
Experience of disruptive behaviors					
Yes	80 (47.1%)	90 (52.9%)	0.212 (0.136–0.329)	0.229 (0.141–0.372)	0.000*
No	193 (80.8%)	46 (19.2%)		**1**	
Age					
20–29	109 (65.7%)	57 (34.3%)	1.328 (0.727–2.426)	1.049 (0.494–2.228)	0.900
30–39	128 (70.3%)	54 (29.7%)	1.646 (0.902–3.003)	1.381 (0.661–2.886)	0.391
40–62	36 (59%)	25 (41%)		**1**	
Attitude towards inter-professional collaboration with physicians					
Favorable	178 (76.1%)	56 (23.9%)	2677 (1.754–4.085)	3.085 (1.862–5.044)	0.000*
Unfavorable	95 (54.3%)	80 (45.7%)		**1**	
Occupational status in the hospital					
Staff nurse and midwife	267 (67.9%)	126 (32.9%)	3.532 (1.256–9.933)	8.175 (2.396–27.891)	0.001*
Nurse and midwife head	6 (37.5%)	10 (62.5%)		**1**	
Time needed for collaboration					
Yes	173 (68.9%)	78 (31.1%)	1.286 (0.846–1.957)	0.768 (0.458–1.291)	0.320
No	100 (63.3%)	58 (36.7%)		**1**	

Variables whose *p*-values written with “*” are statistically significant

“**1**” indicates the category used as a constant in multivariable logistic regression

Again, staff nurses and midwives ((ordinary staffs, those who have no managerial role)) were 8.18 times more likely to have satisfactory inter-professional collaboration with physicians compared with head nurses and midwives (*p* = 0.001, AOR = 8.18, CI: 2.396–27.891). The other associations are illustrated in table three below.

All four statistically significant factors had strong association with participant’s inter-professional collaboration with physicians.

## Discussion

In this study, it could be thought that there is good inter-professional collaboration between nurses and midwives with physicians. But, the proportion of those with unsatisfactory IPC could not be undermined. Again, three of the four significant factors affecting this collaboration were those related to the individual participants.

This study indicates that more than six out of 10, 273 (66.7%) respondents (234 (65.5%) of nurses and three fourth, 39 (75%) of midwives) had satisfactory inter-professional collaboration.

Relatively similar findings were obtained in studies done in Saudi Arabiya (59.5%), Canada (moderate collaboration obtained) and Tigray Ethiopia (60%) [25–27]. But this finding is inconsistent with (showed better collaboration) than studies done in the Netherlands (38.4%), Iran (10.5%) and Ethiopia (41%) [10, 12, 13]. Better collaboration than this study was obtained in a study conducted in Malaysia (91.8%) [24].

This inconsistency may be due to a study setting difference as the Netherlands ‘study was done only in obstetrical care and the study done in Ethiopia was undertaken in two separate hospitals and this could make difference in the collaborative experience of participants. It may also be a result of the difference in time of study conducted as present-day professionals are educated and trained with curriculums and programs that encourage interactive and collaborative working than previous education and training programs that are based on hierarchical models. It could again be attributable to sample size variation.

The analysis of this study also showed that relationship with physicians was found to be a significant factor (AOR, 3.478 (2.077–5.825). This finding is in line with the study done in Tigray, Ethiopia [25]. However, relationship was not a significant factor affecting the nurse’s collaboration with physicians with a study done in Malaysia [24].

This variation may be due to a difference in the study area and study time. It is because nurses and midwives who are working in advanced and more educated areas may have a better understanding of the benefits of good relationship. Again, present-day professionals may be considered better in their experience of relationship and collaboration as they are trained in an improved and interactive manner than before. Also, it might have resulted from socio-cultural variation.

It is worth noting in this study that 170(41.5%) of participants experience at least one disruptive behavior with physicians. Moreover, the experience of disruptive behavior with physicians was also found to decrease the participant’s inter-professional collaboration with physicians (AOR, 0.229 (0.141–0.372). Almost nearer finding was obtained in an Iranian study [11]. But, this finding was inconsistent with (lower than) the study done in West Coast, the United States of America (USA) as most of (92.5%) 0f the respondents mention that they encounter disruptive behaviors though it was not mentioned as a significant factor [28].

The possible reason for this may be study time variation as described earlier that health professionals nowadays are trained together and in an interactive manner that could help them reduce such behaviors later on in working areas. The variation may also be due to study setting disparity as the study under comparison was conducted on 84 different hospitals both in the rural and urban areas hence incorporating participants of different cultural and work experiences.

The other finding of this study was that participants with favorable attitudes towards interprofessional collaboration with physicians were better to collaborate compared to those with unfavorable attitudes towards interprofessional collaboration with physicians (AOR, 3.085 (1.862–5.044). A similar finding was obtained in a study conducted in Palestine [14]. But, this finding is not in line with the finding of the study done in Malaysia and Egypt [16, 24].

This variability could have resulted from a difference in socio-cultural variation as the studies under comparison are conducted in a greatly different environment than this study and this could directly or indirectly affect their attitude. It may also be due to a variation in sample size as both comparing studies were conducted on smaller samples compared to the case of this study thus limiting the variability of responses obtained.

One more important result of this study was that staff nurses and midwives were better to collaborate with physicians compared with head nurses and midwives (AOR, 8.175 (2.396–27.891). This may be as a result that power struggle could be common between those at the managerial roles and physician thus affecting their collaboration. Moreover, staff nurses and midwives are more frequent to meet with physicians and hence may perform many activities together. But, it was difficult to set comparisons to this crucial verdict as all the reviewed works of literature did not consider this issue.

## Conclusion

In this study, the majority of the participants had a satisfactory inter-professional collaboration with physicians without undermining that a considerable figure of them also had unsatisfactory inter-professional collaboration. It is also revealed that there was no significant difference between midwives and nurses in their inter-professional collaboration with physicians.

Moreover, the result of this study showed that among many possible factors under consideration, only four of them including relationship of participants with physicians, respondent’s attitude towards collaborating with physicians, the experience of disruptive behaviors and occupational status in the hospital were found to significantly affect participant’s collaborative work with physicians. Based on the findings of the study, the following recommendations are forwarded.

Jimma University specialized teaching hospital should try to build a conductive and smooth working environment for nurses and midwives through improving their collaboration with physicians. This could be achieved by conducting an environment scan on overall teamwork status and through preparing ongoing training, seminars, and workshops on the benefits of inter-professional collaboration.

Jimma University should help improve collaborative work between nurses and midwives with physicians. This could be achieved by providing related training, workshops, and seminars on the issue.

Jimma University should also design ways that promote interaction between medical students with nursing and midwifery students like strengthening the team training program which could help these future professionals understand each other’s roles and responsibilities. This will help them better collaborate later on in the work environment.

Future researchers are recommended to conduct a qualitative study to have a detailed understanding of the problem by including the experience from the side of physicians too.

## Data Availability

The datasets used for this study could be deposited in publicly available repositories where appropriate and upon reasonable request. All relevant raw data supporting the findings and conclusions of this study can also be freely available from the corresponding author through email “enemelkamu@gmail.com “or with other means to any scientist wishing to use them for non-commercial purposes without breaching participant confidentiality upon reasonable request. There will not be any concern on ethical aspect for this as participant data was de-identified.

## Abbreviations

AOR: Adjusted Odds Ratio
BSc: Bachelor Science
CI: Confidence Interval
COR: Crude Odds Ratio
HSDP: Health Sector Development plan
IPC: Inter- Professional Collaboration
IQR: Inter- Quartile Range
JSANPC: Jefferson Scale of Attitudes towards Nurse- Physician Collaboration
SD: Standard Deviation
SPSS: Statistical Packages for Social Sciences
WHO: World Health Organization
